# Immediate voluntary activation deficits following submaximal eccentric contractions of knee extensors are associated with alterations of the sense of movement

**DOI:** 10.1038/s41598-022-06081-2

**Published:** 2022-02-11

**Authors:** Flavio Da Silva, Serge S. Colson, Firas Zghal, Frédéric Chorin, Olivier Guérin, Florian Monjo

**Affiliations:** 1 Université Côte d’Azur, Laboratoire Motricité Humaine Expertise Sport Santé (LAMHESS, UPR 6312), Ecole Universitaire de Recherche HEALTHY: Ecosystèmes des Sciences de la Santé, Campus STAPS – Sciences du Sport, 261, Boulevard du Mercantour, 06205 Nice Cedex 03, France; 2 Université Côte d’Azur, CHU, Nice, France; 3 Université Côte d’Azur, CNRS, Inserm, IRCAN, Nice, France

**Keywords:** Neurophysiology, Motor control, Somatosensory system

## Abstract

The mechanisms underlying movement sense alterations following repeated eccentric contractions remain unclear. This study concomitantly investigated the effects of unilateral eccentric contractions on movement sense and on neuromuscular function at the knee before, immediately after (POST), 24 (POST24) and 48 (POST48) h after the exercise. Twelve participants performed sets of submaximal knee extensors (KE) eccentric contractions until a 20% decrease in maximal voluntary isometric contraction (MVIC) torque was reached. Threshold to detect passive movement (TTDPM) tasks were used to assess movement sense during both knee flexion (TTDPM_FLEX_) and extension (TTDPM_EXT_). KE fatigability was assessed using the interpolated twitch technique. TTDPM values expressed in seconds and the percentage of unsuccessful trials only increased at POST during TTDPM_FLEX_ and TTDPM_EXT_. The 20%-MVIC decrease was associated with significant decreases in voluntary activation level (− 12.7%, *p* < 0.01) and potentiated doublet torque at 100 Hz (− 18.1%, *p* < 0.001). At POST24, despite persistent reductions of maximal voluntary and electrically evoked torques associated with increased perceived muscle soreness, TTDPM values and the percentage of unsuccessful trials returned to baseline values. Consequently, movement sense alterations were only observed in the presence of voluntary activation deficits, suggesting that some exercise-induced central alterations may affect the somatosensory function.

## Introduction

During exercise, the repetition and/or maintenance of muscle contractions results in a transient reduction in the muscle’s ability to produce force or power^1^. This transient decline, which refers to performance fatigability, is influenced by complex interactions between peripheral and central factors, involving contractile function and muscle activation alterations^2,3^. In addition, performance fatigability has been associated with alterations in proprioceptive senses, such as the sense of effort^4^ and the sense of force^5^.

Proprioceptive senses also encompass kinesthesia, which refers to the conscious sensations of body positions and movements^6^, whether generated in a voluntary or passive manner^7^. Kinesthesia, by providing body representation in space, obviously plays a fundamental role in sensorimotor control and especially relies on signals stemming from muscle spindles^8^. These intramuscular sensory structures are connected to force-generating extrafusal fibers and are sensitive to muscle lengthening and shortening. More precisely, group Ia afferents inform the central nervous system (CNS) of length change and rate of length change^8^, whereas secondary endings of group II afferents are length sensors^9^. Thereby, secondary endings seem to play a major role in the position sense, while primary endings predominantly play a role in the movement sense. In addition, previous studies have highlighted that skin stretch receptors also contribute to movement sense through slow adapting type II receptors served by Ruffini endings^10^. Finally, joint mechanoreceptors contribute to kinesthesia predominantly in extreme angular positions^11^.

As performance fatigability is inherent to exercise and sports performance, investigating its effects on kinesthesia is of major interest. In the literature, particular focus has been given to the effects of eccentric exercise on kinesthesia because this contraction mode is known to induce muscle damage to force-generating extrafusal fibers^12,13^. Muscle damage has been assumed to propagate to muscle spindles and lead to kinesthetic alterations^8^. Although no significant alterations of muscle spindle firing rates in the presence of eccentric-induced damage were reported in animal experimentation^14^, significant position sense alterations following repeated eccentric contractions were, however, reported in human studies^15–19^. The origin of the kinesthetic alterations thus remains unclear, despite the recent research pointing to the implication of central alterations^17–19^. Indeed, Da Silva et al.^19^ observed a significant correlation between knee position sense alterations and decreases in voluntary activation levels (VA) after submaximal eccentric contractions of knee extensors (KE). This suggests that the central alterations induced by the repetition of eccentric contractions might affect the integration and processing of muscle spindle afferents, accounting for position sense alterations.

Contrary to position sense assessment, and as highlighted by Proske^20^, the effects of repeated eccentric contractions on movement sense have been overlooked. Given its central role in sensorimotor control, investigating the effects of eccentric exercise on movement sense is of interest in the field of applied physiology and sports sciences. Experimentally, two psychophysical tasks have been used to assess movement sense: contralateral tracking tasks^21^ and threshold to detect passive movement (TTDPM) tasks^22–24^. TTDPM tasks have been shown to be reliable and valid to evaluate movement sense^25^. During TTDPM tasks, upper or lower limbs are passively moved at very slow angular velocities (i.e., usually below 0.5° s^−1^) and participants are instructed to press a hand-held button when they perceive a joint movement^26^. TTDPM values, expressed in seconds, represent the time lapse between the beginning of the joint movement and its detection. Because of the lack of studies and the different tasks used, debate continues as to whether eccentric contractions affect movement sense immediately and over the days following the exercise. Moreover, experiments which have shown movement sense alterations^23,24^ did not seek to determine the underlying mechanisms.

In this context, knee movement sense was evaluated with TTDPM tasks before and immediately after repeated unilateral KE eccentric contractions, as well as 24, and 48 h post-exercise. To the best of our knowledge, this is the first study to concomitantly evaluate KE neuromuscular function and movement sense, the aim being to better understand the mechanisms underlying movement sense alterations following repeated eccentric contractions. Based on recent observations showing correlations between position sense alterations and VA decreases^19^, we hypothesized that movement sense alterations would only be observed in the presence of VA deficits, i.e., only immediately after the eccentric exercise.

## Methods

### Participants

Based on previous studies that reported VA decreases^27^ and movement sense alterations^24^ following eccentric exercise of the KE, we used GPower (version 3.1.9.4; Kiel University, Kiel, Germany) and estimated that at least seven participants would be required to find a significant effect for repeated measures within-subject analysis with the power set at 0.95. Accordingly, twelve physically active participants [eight males (age: 26.1 ± 4.4 years; body mass: 79.4 ± 7.8 kg; height: 181.6 ± 5.5 cm) and four females (age: 24.0 ± 1.4 years; body mass: 58.0 ± 4.7 kg; height: 168.8 ± 4.7 cm)] volunteered to participate in this study. Based on our study’s sample size, a sensitivity power analysis with an α of 0.05 and a power of 0.95 resulted in a large effect of *f* = 0.45. Participants recruited had been free from knee neuromuscular injuries for at least six months and had never undergone any kind of knee surgery. They were fully informed of the testing procedures and gave their informed written consent at their arrival to the laboratory before any testing. The local research ethics committee approved the study and the experimental procedures were conducted in accordance with the latest version of the Declaration of Helsinki (2013), with the exception that the trial was not registered in a trial database.

### Experimental protocol

**Figure 1:**
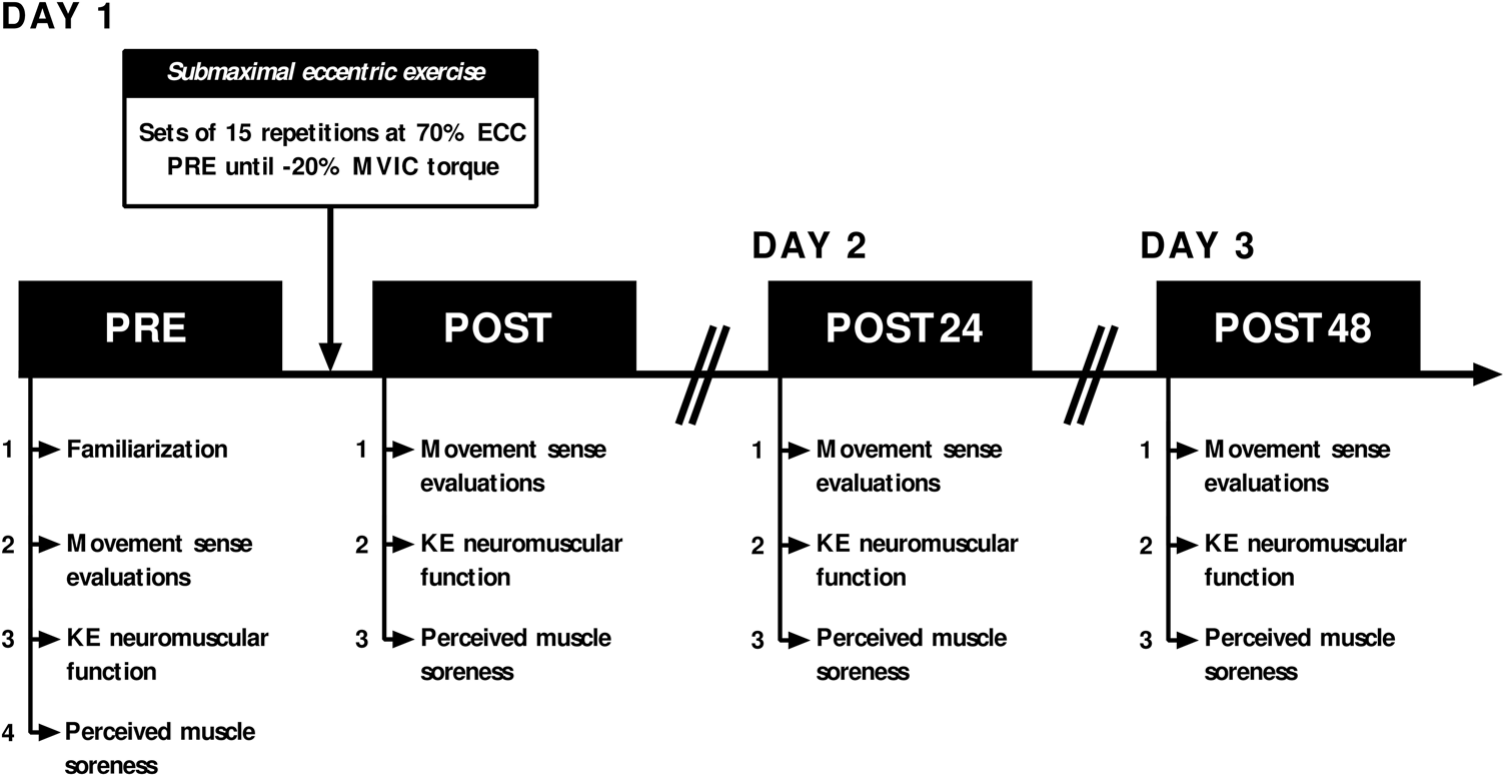
Overview of the experimental protocol. Neuromuscular and psychophysical evaluations were performed before (PRE) and immediately after (POST) a unilateral fatiguing eccentric exercise at the knee. PRE evaluations were similarly performed 24 (POST24) and 48 (POST48) hours after the eccentric exercise, without the familiarization period. Please refer to the text for further explanations. *KE* Knee extensors*, ECC* Maximal voluntary eccentric contraction*, MVIC* Maximal voluntary isometric contraction.

To assess immediate and long-lasting effects of a submaximal eccentric exercise on the knee movement sense and on the KE neuromuscular function, participants were required to perform three experimental sessions separated by 24 h (Fig. 1). Neuromuscular and psychophysical evaluations were performed at four time points: before (PRE), immediately after (POST), 24 (POST24) and 48 (POST48) hours after a submaximal eccentric exercise. The warm-up protocol consisted of three sets interspaced by 1-min of continuous KE concentric and eccentric contractions. Participants were required to progressively increase the intensity of contractions (i.e., from 50 to 90%) with a gradual decrease in angular velocity. Participants first performed 12 contractions at 90° s^−1^, then 8 contractions at 60° s^−1^, and finally 4 contractions at 30° s^−1^.

### Submaximal eccentric exercise

Participants performed a unilateral submaximal exercise on their right lower limb that consisted of several sets of 15 submaximal eccentric contractions. The contraction intensity was set at 70% of the maximal voluntary eccentric contraction (ECC) measured in PRE. A horizontal line provided on the screen served as visual feedback of the target intensity. The angular velocity was set at of 30° s^−1^ with a range of motion of 100°, from 5° to 105° (0° = fully extended). After each KE eccentric contraction, the limb was passively moved into full extension at an angular velocity of 90° s^−1^. At the end of each set of 15 contractions, participants were asked to perform a maximal voluntary isometric contraction (MVIC) without electric nerve stimulations to evaluate the level of MVIC force loss. If the MVIC decrement was less than 20% of the PRE value, a new set of 15 eccentric contractions was performed. Sets were repeated until participants reached a force loss representing a 20% MVIC decrement. This target value was chosen to match the force decrements observed following ecological exercises such as participating in soccer games^28^ or downhill running^29^. An additional set of seven eccentric contractions with similar parameters (i.e., 70% of ECC PRE at 30° s^−1^) was performed in POST between the two tested directions for movement sense evaluations to maintain the target 20% MVIC loss.

### Neuromuscular function assessments

#### Torque measurements

Participants were placed on an isokinetic dynamometer (Biodex System 4, Biodex Medical Systems, Shirley, NY, USA) showing good reliability, to measure voluntary and electrically evoked torques^30^. They sat on the dynamometer with a 95° hip angle and the knee joint axis aligned with the axis of the dynamometer lever arm. The lever arm was fixed to the dynamometer motor axis and attached 2 cm above the external malleolus of the participants’ right limb. To avoid any movements during the protocol, participants were firmly strapped with belts over the chest and hips. Torque measurements were recorded at a sampling rate of 500 Hz using commercially available software (Acknowledge 4.1 Biopac Systems, Inc., Goleta, CA,, USA). To evaluate and control participants’ KE fatigability, MVIC and ECC were performed. The knee angle during MVICs was set at 60° of knee flexion, while ECCs were performed over the full range of motion (i.e., from 5 to 105° of knee flexion) at an angular velocity of 30° s^−1^. Each maximal contraction, whether isometric or eccentric, was interspaced by a 1-min rest. The mean value of the torque signal was calculated over a 500-ms window around the highest torque value for MVICs, while highest peak torque values were recorded for ECC. At PRE, POST24, and POST48, participants performed two MVICs and ECCs. Only the best value of these contractions was retained for data analysis. Immediately after the eccentric exercise (i.e., at POST), only one MVIC and one ECC were performed. At the very end of POST condition, another MVIC (MVIC_END_) was performed to ensure that the target 20%-MVIC torque decrement was maintained.

#### Contractile function and voluntary activation of the quadriceps

The effects of the eccentric exercise on both central and peripheral factors of KE fatigability were investigated using the interpolated twitch technique during MVICs^31^. For this purpose, the cathode, a self-adhesive surface electrode (diameter: 10 mm, Ag–AgCl, Contrôle-Graphique S.A., Brie-Comte-Robert, France) was positioned on the femoral triangle. The anode electrode (self-adhesive rectangular electrode, 5 cm × 9 cm—Stimex, Wetzlar, Germany) was placed at the level of the great trochanter. Placements of cathode and anode electrodes were marked on the skin to ensure a similar repositioning at POST24 and POST48. Rectangular pulses (400 V maximum voltage and 1 ms duration) were delivered at the level of the femoral nerve by an electrical stimulator (Digitimer Stimulator DS7, Digitimer Ltd., Hertfordshire, UK) to induce maximal mechanical twitches (i.e., Tw) and compound muscle action potentials (i.e., maximal M-wave, M_MAX_) of KE at rest. To ensure adequate assessment of central and peripheral components of KE neuromuscular function^32^, the stimulation intensity (135.7 ± 28.5 mA) was set to 120% of the optimal stimulation intensity which generated maximal Tw and M_MAX_. MVICs were associated with an interpolated 100-Hz paired stimuli (Dt_sup_) over MVIC plateaus and two paired electrical stimulations at a frequency of 10 Hz (Dt_10 Hz_) and 100 Hz (Dt_100 Hz_) after the contraction. Interpolated 100-Hz paired stimuli were used to evaluate KE VA. Then, Dt_sup_ and Dt_100 Hz_ were used to calculated VA according to the formula proposed by Merton^33^:

$$\mathrm{VA }(\mathrm{\%}) = \left(1-\left(\frac{{Dt}_{sup}}{{Dt}_{100 Hz}}\right)\right)*100$$

Dt_100 Hz_, Dt_10 Hz_ and Dt_10 Hz_-to-Dt_100 Hz_ ratio (Dt_10 Hz_/Dt_100 Hz_) measurements were used for further analysis.

#### Surface electromyography (EMG) recordings

Before the placement of EMG surface electrodes, participants’ skin was prepared by shaving, lightly abrading and cleaning with alcohol to reduce impedance at the skin–electrode interface below 5 kΩ. Electrical muscle activity was recorded using six pairs of EMG surface electrodes (Ag/AgCl, diameter = 10 mm; inter-electrode distance = 20 mm; Contrôle-Graphique, Brie-Comte-Robert, France) placed according to the SENIAM recommendations^34^ on the rectus femoris (RF), the vastus lateralis (VL), the vastus medialis (VM) and the biceps femoris (BF) of the exercised limb. A reference electrode was also positioned on the right lateral tibial condyle. To ensure a similar repositioning over the consecutive days (i.e., at POST24 and POST48), each electrode placement was marked on the skin. EMG signals were amplified using the Biopac MP150 system (Biopac Systems, Inc., Goleta, CA,, USA) within a bandwidth frequency ranging from 10 to 500 Hz (common mode rejection ratio = 110 dB, Z Input = 1000 MΩ, gain = 1000). During voluntary and electrically evoked contractions, EMG signals were recorded at a sampling frequency of 2 kHz. Root mean square (RMS) values of KE EMG of each muscle were computed over a 500-ms period during MVICs and ECCs and normalized to the respective M_MAX_ value (i.e., average of the three responses) recorded at each time point^31^. The level of antagonist coactivation during maximal voluntary torque production was calculated using the ratio between the BF RMS value computed over the same period and the sum of the RMS/M_MAX_ values of RF, VL and VM muscles^35^.

### Psychophysical evaluations

#### Movement sense evaluations

Evaluations of knee movement sense were performed on the dynamometer using TTDPM tasks during both knee flexion (TTDPM_FLEX_) and extension (TTDPM_FXT_). Participants seated in the position described above and all audiovisual cues were eliminated as participants were blindfolded and wore noise-canceling headphones. A cuff was placed on participants’ leg to attenuate any tactile cues related to the displacement of the dynamometer lever arm^36^. Only the right exercised limb was tested and directions (i.e., TTDPM_FLEX_ and TTDPM_EXT_) were randomized in a counterbalanced order between participants. One trial was performed as follows: (i) the limb was passively moved and supported by the dynamometer lever arm to a starting angle of 30° of knee flexion, (ii) an experimenter touched the limb of the participant to inform him/her that the movement could be initiated randomly within 30 s, (iii) the dynamometer arm moved passively at a constant very slow speed (i.e., angular velocity of 0.25° s^−1^)^24^ into one of the tested directions (i.e., TTDPM_FLEX_ and TTDPM_EXT_), and (iv) the participant pressed a button placed in his/her left hand as soon as he/she felt sensation of movement or a change in the initial position of their leg. To limit learning effects, participants were familiarized with TTDPM tasks before the start of the protocol. During familiarization, participants performed five trials per tested direction as described previously.

Throughout the experimental protocol, knee angles and time values were recorded over three trials for each tested direction (i.e., TTDPM_FLEX_ and TTDPM_EXT_) at each time point (i.e., PRE, POST, POST24 and POST48). Instead of using typical angle errors, TTDPM values were computed as the time (in seconds) between movement initiation and the moment when participants pressed the button. Only the best trial of each set per condition (i.e., the trial with the lowest TTDPM values in seconds) was used for further analysis. Moreover, in some trials, participants felt that their limb was moving while it was not (i.e., they pressed the button while the lever arm had not started to move). To consider these failed trials, a statistical analysis of the distribution between successful and unsuccessful trials was performed using binary encoding. For this purpose, we assigned values of 1 to successful trials (TTDPM of ≤ 2.5°, i.e., less than or equal to 10 s to detect the movement) and 0 to unsuccessful trials (TTDPM of > 2.5°, i.e., more than 10 s to detect the movement plus the failed trials). All trials were considered for statistical analyses.

#### Perceived muscle soreness (PMS)

Participants were asked to estimate the level of their PMS at each time point using a 0–10 numerical rating scale (0: no pain at all; 10: worst pain ever). For this purpose, a constant pressure of 25 N was applied next to the proximal EMG electrode of the corresponding KE muscle (i.e., RF, VL and VM) with a cylindrical object (0.5 cm diameter)^37^. PMS scores of the RF, VL and VM were averaged across muscles at each time point for further statistical analysis.

### Statistical analyses

KE fatigability and PMS parameters were analyzed using one-way analyses of variance (ANOVAs) with repeated measures (PRE vs. POST vs. POST24 vs. POST48). ANOVAs were performed using Statistica software version 8.0 (StatSoft Inc., Tulsa, OK, USA). TTDPM values of the best trial of each set were submitted to a 2-direction (TTDPM_FLEX_ vs. TTDPM_EXT_) × 4 time points (PRE vs. POST vs. POST24 vs. POST48) repeated measure ANOVA. Data were first screened for normality of distribution and homogeneity of variances using the Kolmogorov–Smirnov test and the Bartlett test. When ANOVAs displayed significant main or interaction effects, a Tukey HSD post-hoc test was applied. Effect sizes were determined from partial eta square values (η^2^p) with interpretation thresholds fixed at 0.01, 0.08, 0.26, and 0.50 for small, moderate, large, and very large effect sizes, respectively. The values are reported in tables and figures as means ± standard deviations (SD). Significance level was set at *p* < 0.05 for all statistical analyses.

The effects of the eccentric exercise on the distribution between successful and unsuccessful trials were studied using a generalized linear mixed-effect model (GLMM) with binomial distribution and logit link function performed using IBM SPSS Statistics software for Windows version 27 (IBM Corp., Armonk, NY, USA). GLMMs are an extension of linear mixed models that allow binary repeated measures to be statistically analyzed^38^. Models with and without inclusion of a random slope were compared using the Akaike information criterion (AIC) and the model with the lowest score was retained as recently recommended^39^. Thus, the GLMM used included the following fixed effects: the time points (PRE vs. POST vs. POST24 vs. POST48), the tested direction (TTDPM_FLEX_ vs. TTDPM_EXT_) and the trial order within sets (to control for order effects within measures). When the model displayed significant main or interaction effects (*p* < 0.05), Sidak adjusted pairwise comparisons were performed.

### Ethics declarations

Approval was obtained from the ethics committee of Université Côte d’Azur. This study was conducted in accordance with the latest version of the Declaration of Helsinki.

### Consent to participate

Informed consent was obtained from all individual participants included in the study.

### Consent for publication

Participants signed informed consent regarding publication of their data.

## Results

During the submaximal eccentric exercise, an average eccentric peak torque value of 251.3 ± 81.1 Nm was reached, and participants performed 76 ± 34 contractions to reach the target of a 20% MVIC decrease.

### Neuromuscular function parameters

#### Maximal voluntary torque production

**Figure 2:**
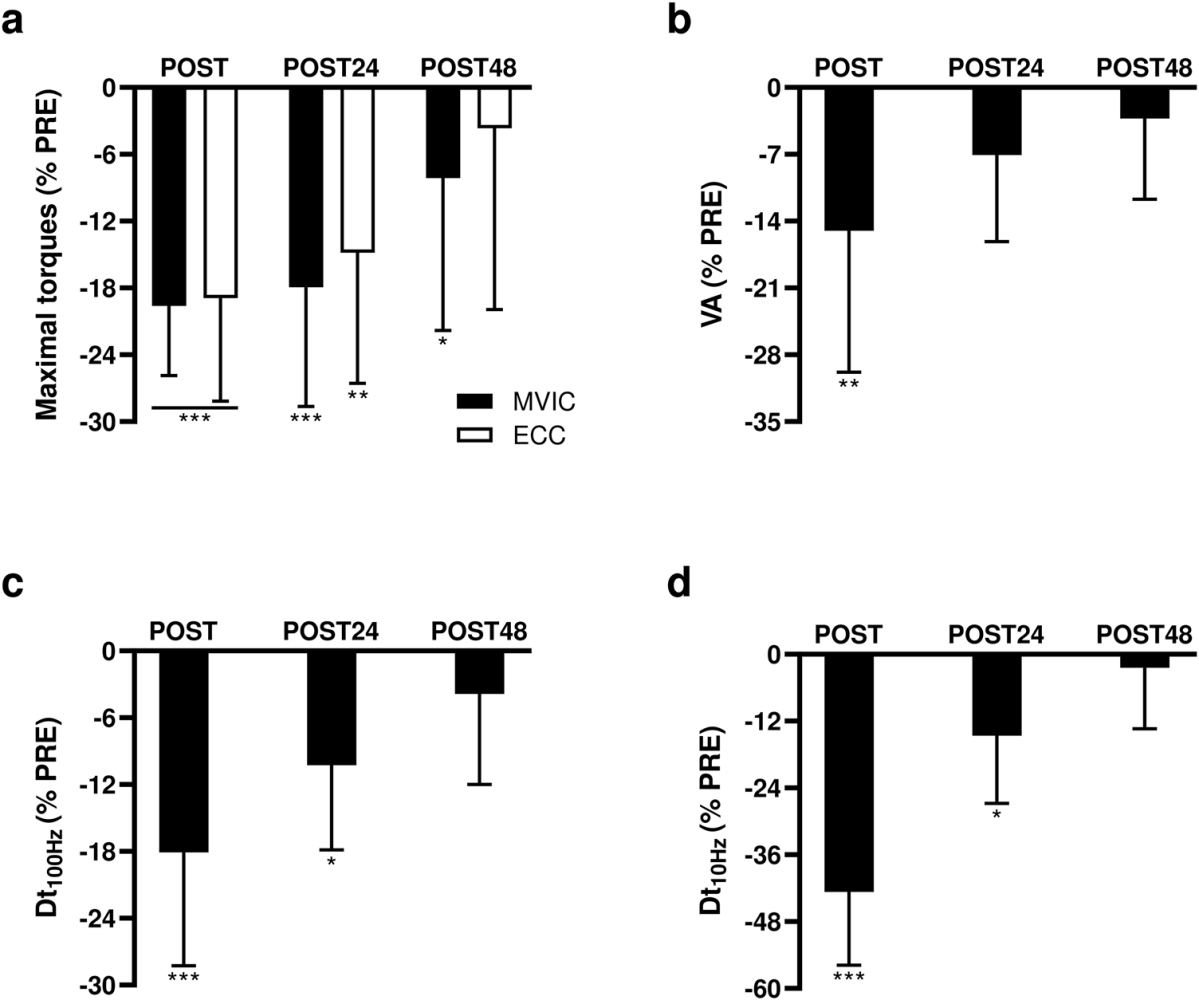
Effects of the submaximal eccentric exercise on knee extensor’s neuromuscular function. Percent changes from pre-exercise values in (**a**) maximal voluntary torque production (MVIC in black bars and ECC in white bars), (**b**) VA , (**c**) Dt_100 Hz_ and (**d**) Dt_10 Hz_. *, ** and ***Significantly different from PRE values at *p* < 0.05, *p* < 0.01 and *p* < 0.001, respectively*. MVIC* Maximal Voluntary Isometric Contraction, *ECC* Maximal voluntary eccentric contraction, *VA* Voluntary Activation level, *Dt*_*100 Hz*_ potentiated doublet amplitude at 100 Hz, *Dt*_*10 Hz*_ potentiated doublet amplitude at 10 Hz. The statistical analyses of this figure were performed using Statistica software.

A significant main effect of the time point was found for MVIC (F_3, 33_ = 13.3, *p* < 0.001, η^2^p = 0.55) and ECC (F_3, 33_ = 12.9, *p* < 0.001, η^2^p = 0.54). MVIC and ECC torques were significantly impaired following the eccentric exercise (*p* < 0.001; Table 1 and Fig. 2a). At POST24, MVIC and ECC torques remained significantly lower compared to PRE values (*p* < 0.01), but were no longer different from PRE values at POST48 (*p* > 0.12). MVIC_END_ (189.1 ± 69.1 N.m) was not statistically different from MVIC measured at POST (211.5 ± 66.2 N.m, *p* = 0.07).

**Table 1:** Mean values ± SD of neuromuscular function parameters at each time point.

	PRE	POST	POST24	POST48
MVIC (N m)	263 ± 77	212 ± 66***	218 ± 74***	242 ± 80
ECC (N m)	359 ± 116	293 ± 107***	309 ± 118**	346 ± 126
VA (%)	89 ± 8	76 ± 17**	83 ± 12	86 ± 12
Dt_100 Hz_ (N m)	99 ± 26	83 ± 29***	91 ± 29*	95 ± 25
Dt_10 Hz_ (N m)	93 ± 25	55 ± 23***	82 ± 30*	91 ± 26
Dt_10 Hz_/Dt_100 Hz_ (%)	94 ± 8	65 ± 9***	89 ± 12	95 ± 9
RF (mV)	5.6 ± 1.5	5.4 ± 1.5	5.7 ± 1.8	5.4 ± 1.8
VL (mV)	5.5 ± 4.1	5.4 ± 3.9	5.8 ± 4.0	5.7 ± 3.7
VM (mV)	5.1 ± 2.0	4.9 ± 2.3	4.5 ± 1.6	4.6 ± 1.4
**During MVIC**				
RF EMG RMS/M_MAX_	0.06 ± 0.02	0.06 ± 0.02	0.05 ± 0.02	0.06 ± 0.02
VL EMG RMS/M_MAX_	0.07 ± 0.03	0.06 ± 0.02	0.07 ± 0.03	0.07 ± 0.02
VM EMG RMS/M_MAX_	0.08 ± 0.05	0.07 ± 0.03	0.08 ± 0.04	0.07 ± 0.03
Coactivation	0.21 ± 0.17	0.22 ± 0.15	0.21 ± 0.19	0.19 ± 0.12
**During ECC**				
RF EMG RMS/M_MAX_	0.07 ± 0.02	0.06 ± 0.02	0.07 ± 0.02	0.07 ± 0.02
VL EMG RMS/M_MAX_	0.08 ± 0.03	0.06 ± 0.02	0.07 ± 0.03	0.08 ± 0.03
VM EMG RMS/M_MAX_	0.08 ± 0.04	0.08 ± 0.03	0.08 ± 0.03	0.08 ± 0.03
Coactivation	0.17 ± 0.04	0.32 ± 0.48	0.18 ± 0.06	0.22 ± 0.18

Bold values are significant. *, ** and ***Significantly different from PRE values at *p* < 0.05, at *p* < 0.01 and at *p* < 0.001, respectively. *MVIC* Maximal Voluntary Isometric Contraction, *ECC* maximal voluntary eccentric contraction, *VA* Voluntary Activation level, *Dt*_*100 Hz*_ potentiated doublet amplitude at 100 Hz, *Dt*_*10 Hz*_ potentiated doublet amplitude at 10 Hz, *Dt*_*10 Hz*_*/Dt*_*100 Hz*_ low-to-high doublet frequency ratio, *M*_*MAX*_ Maximal M-wave amplitude, *RF* Rectus Femoris, *VL* Vastus Lateralis, *VM* Vastus Medialis, *EMG RMS/M*_*MAX*_ Root Mean Square values of the electromyographic recordings normalized to M_MAX_. The statistical analyses in this table were performed using Statistica software.

#### Electrically evoked torques and M_MAX_

A significant effect of the time point was found for Dt_100 Hz_ (F_3,33_ = 13.4, *p* < 0.001, η^2^p = 0.55), Dt_10 Hz_ (F_3,33_ = 50.3, *p* < 0.001, η^2^p = 0.82) and Dt_10 Hz_/Dt_100 Hz_ (F_3,33_ = 44.9, *p* < 0.001, η^2^p = 0.80). At POST, Dt_100 Hz_, Dt_10 Hz_ and Dt_10 Hz_/Dt_100 Hz_ values were significantly reduced (*p* < 0.001; Fig. 2c and 2d). The eccentric exercise also induced persistent significant reductions of Dt_100 Hz_ and Dt_10 Hz_ at POST24 (*p* < 0.05; Fig. 2c,d). Dt_100 Hz_ and Dt_10 Hz_ were no longer different from PRE values at POST48 (*p* > 0.47).

The fatiguing eccentric exercise had no effect on RF M_MAX_ (*p* = 0.31, η^2^p = 0.10), VL M_MAX_ (*p* = 0.13, η^2^p = 0.15) or VM M_MAX_ (*p* = 0.48, η^2^p = 0.07) values (Table 1).

#### VA and EMG recordings during maximal torque productions

A significant effect of the time point was found for VA (F3,33 = 6.0, *p* < 0.01, η^2^p = 0.35). VA values were significantly reduced at POST only (*p* < 0.01; Table 1 and Fig. 2b).

Whether during MVIC or ECC, the eccentric exercise did not induce significant changes of RF EMG RMS/M_MAX_, VL EMG RMS/M_MAX_, VM EMG RMS/M_MAX_ values (*p* > 0.07, η^2^p < 0.20; Table 1). Additionally, the computed antagonist coactivation values during MVIC and ECC were unaffected by the eccentric exercise (*p* > 0.48, η^2^p < 0.08; Table 1).

### Psychophysical parameters

#### Movement sense

##### TTDPM

**Figure 3:**
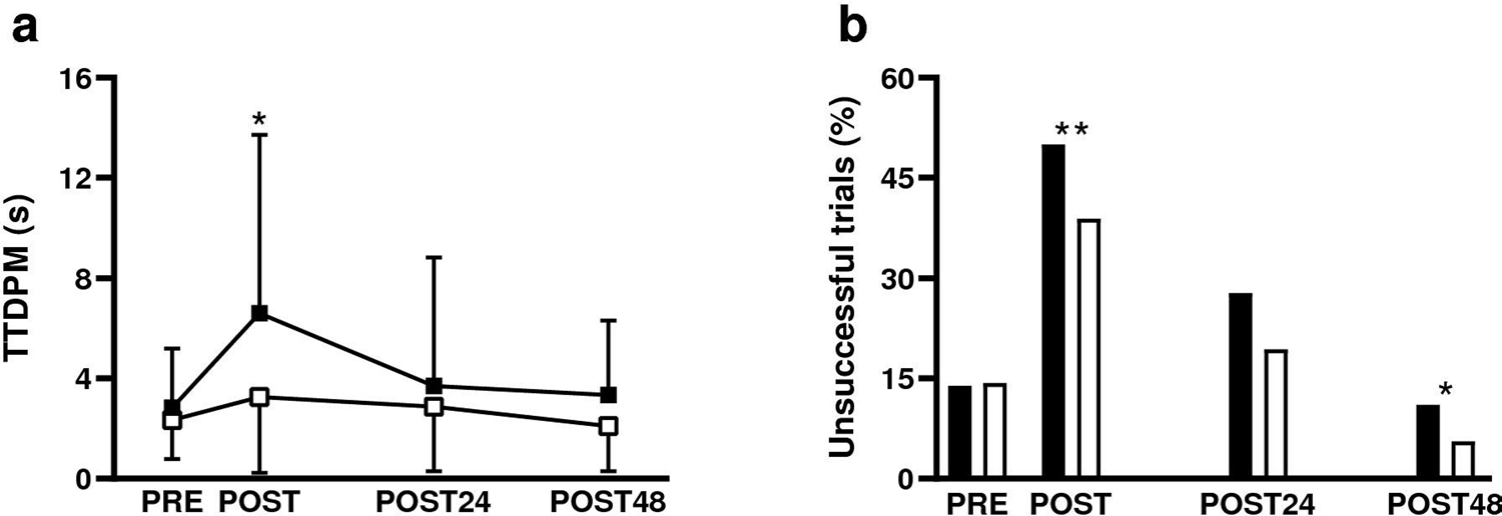
Effects of the eccentric exercise on knee movement sense. (**a**) TTDPM best trial values and (**b**) percentages of unsuccessful trials during TTDPM tasks before (PRE), immediately after (POST), and 24 (POST24) and 48 (POST48) hours after the eccentric exercise. ^*^ and ^**^Significantly different from PRE values at p < 0.05 and p < 0.01, respectively. *TTDPM*_*FLEX*_ Threshold To Detect Passive Movement into flexion at 30° of knee flexion, *TTDPM*_*EXT*_ Threshold To Detect Passive Movement into extension at 30° of knee flexion. The statistical analyses of this figure were performed using IBM SPSS software.

A significant effect of the time point was found for TTDPM values (F_3, 33_ = 3.0, *p* < 0.05, η^2^p = 0.21; Fig. 3a) but no direction or interaction effects were observed (*p* > 0.11). Whatever the tested direction, TTDPM values significantly increased at POST compared to PRE (*p* < 0.05). TTDPM values were no longer different from PRE at POST24, and POST48 (p > 0.84).

##### Distribution of successful and unsuccessful trials

The GLMM (performed without random slopes of the time point) showed a significant effect of the time point (F_3, 263_ = 4.5, *p* < 0.01; Fig. 3b and Table 2). However, no significant effects of the tested direction (*p* > 0.99), the trial order (*p* > 0.98) or the interactions between all fixed factors (*p* > 0.75) were found. Similar to the results of the ANOVA, the number of unsuccessful trials significantly increased at POST (*p* < 0.01) and was no longer different from PRE at POST24 (*p* > 0.40). Note that participants made significantly fewer unsuccessful trials at POST48 compared to PRE (*p* < 0.05).

**Table 2:** Number of successful and unsuccessful trials during movement sense evaluations at each time point.

	TTDPM_FLEX_				TTDPM_EXT_			
	PRE	POST	POST24	POST48	PRE	POST	POST24	POST48
**Successful trials**								
TTDPM ≤ 10 s	29	18	26	33	31	12	29	34
**Unsuccessful trials**								
Failed trials	3	5	4	0	1	8	7	2
TTDPM > 10 s	4	13	6	3	4	6	0	0

*TTDPM* Threshold To Detect Passive Movement, *TTDPM*_*FLEX*_ Threshold To Detect Passive Movement into flexion at 30° of knee flexion, *TTDPM*_*EXT*_ Threshold To Detect Passive Movement into extension at 30° of knee flexion*, Failed trials* trials where participants pressed the button while no movement was generated.

#### PMS

A significant effect of the time point was found for PMS scores (F_3,33_ = 6.9, *p* < 0.001, η^2^p = 0.39). PMS scores measured at POST (1.1 ± 1.3) did not differ from PRE values (0.9 ± 0.9, *p* = 0.90), whereas they were significantly higher at POST24 and POST48 (2.2 ± 1.2 and 2.1 ± 1.5, respectively; *p* < 0.01).

## Discussion

The present study was conducted to evaluate immediate and long-lasting effects of repeated KE eccentric contractions on knee movement sense using TTDPM tasks. For this purpose, concomitant evaluations of KE fatigability and movement sense were carried out before, immediately after, and 24 and 48 h after a submaximal eccentric exercise. The purpose of these concurrent evaluations was to better delineate the mechanisms underlying movement sense alterations following repeated eccentric contractions. In line with our assumptions, movement sense alterations were only observed when VA deficits were present, i.e., only immediately after the eccentric exercise. Despite persistent PMS and alterations of electrically evoked torques at POST24, TTDPM values and the number of unsuccessful trials had returned to baseline values, meaning that movement sense alterations were no longer present at this time point.

At POST, the 20%-MVIC decrease was associated with alterations in electrically evoked torques and VA values with different recovery rates in the days following the eccentric exercise (Table 1 and Fig. 2). In the present study, VA deficits were only observed immediately after the eccentric exercise with a 12.7% decrease at POST (*p* < 0.01; Fig. 2b). In contrast, at POST24, significant reductions in Dt_100 Hz_ and Dt_10 Hz_ indicate that alterations in muscle contractile properties were still present (*p* < 0.01; Fig. 2c and 2d). This long-lasting reduction in electrically evoked torques was associated with a significant increase in PMS (*p* < 0.01), and thus likely indicates the presence of muscle damage commonly observed following repeated eccentric contractions^12,13^. Our observations of acute VA decreases and persistent alterations in electrically evoked torques are consistent with previous research on damaging eccentric contractions^40,41^.

To explain the presence of acute movement sense alterations following the eccentric exercise, two distinct hypotheses can be formulated: i) a peripheral hypothesis where eccentric contractions would have altered muscle spindle integrity or ii) a central hypothesis where the integration and/or processing of movement-related sensory inputs at the CNS level would have been altered. It is unlikely that the peripheral hypothesis could account for our observations. First, due to their compliant connections with extrafusal fibers^14^, muscle spindles seem to be protected from eccentric-induced mechanical damage^14^, suggesting that muscle spindle sensitivity remained unchanged following the eccentric exercise. Second, the number of unsuccessful trials also increased during TTDPM_EXT_ tasks at POST (Fig. 3b). In this tested direction, movement sense mainly relied on signals arising from muscle spindles of the knee flexors which were not submitted to the damage caused by repeated eccentric contractions. This reinforces the idea that the disturbance of movement sense was not due to alterations of muscle spindle integrity.

Regarding the central hypothesis, movement sense was only altered at POST (Fig. 3a and 3b), i.e., in presence of VA deficits (Fig. 2b). Movement sense seemed to be unaffected by the persistent alterations in muscle contractile function and the increased PMS observed the following days (i.e., at POST24 and POST48; Fig. 3a and 3b). Therefore, movement sense alterations followed the time-course of central factor alterations (i.e., VA deficits) related to KE fatigability rather than those of the peripheral alterations. Altogether, these results support the idea that central mechanisms related to VA deficits could have contributed to the acute movement sense alterations reported in the present study. In line with recent propositions^19^, the exercise-induced increase neural flow in groups III and IV afferents at the somatosensory cortex^42^ could have altered the integration and/or processing of Ia and II afferents at the CNS level, contributing to the lowered sensitivity to detect movement-related sensory signals.

Previous research has investigated the immediate and long-lasting effects of repeated eccentric contractions on knee movement sense^23,24^. Consistent with our results, Torres et al.^23^ reported movement sense alterations during TTDPM_EXT_ tasks only immediately after a submaximal eccentric exercise. However, the authors did not investigate central and peripheral factors of performance fatigability. In contrast to our findings, Naderi et al.^24^ observed movement sense alterations up to 48 h after 4 sets of 25 maximal eccentric contractions. In their study, the maximal voluntary torque production capacity was still reduced by 29% 48 h after the eccentric exercise, whereas it had fully recovered in the present study (Table 1 and Fig. 2a). Although VA was not assessed in Naderi et al.’s study, another experiment^43^ observed that 20% torque production capacity reduction was associated with significant VA deficits 48 h after the exercise. Consequently, one could assume that persistent central factor alterations were present in Naderi et al.’s study, thus contributing to the long-lasting movement sense alterations.

From a methodological perspective, the main limitation of the present study is the absence of an independent familiarization session with TTDPM tasks. This might explain the lower number of unsuccessful trials at POST48 compared to PRE (Fig. 3b and Table 2). Nonetheless, this potential limitation had no effect on TTDPM values of the best trial, as shown by the absence of significant differences between PRE and POST48 values (Fig. 3a). Moreover, the effect of the eccentric exercise on the distribution of successful and unsuccessful trials at POST was substantial (Fig. 3a). Therefore, it is highly probable that a familiarization session would not have changed the general tendency of our results. Nonetheless, in view of this possible learning effect, researchers should pay particular attention when familiarizing participants with the tasks used to evaluate movement sense.

Furthermore, for future studies on the effects of exercise on kinesthesia, researchers should consider different methods and statistical approaches. To facilitate the comparisons between studies, Watkins et al.^44^ recently proposed to standardize the task parameters (e.g., passive movement speed). As in the present paper, future studies should use a statistical approach that considers the unsuccessful trials during movement sense assessment and not only typical TTDPM values**.** It would also be important to investigate the effects of eccentric contractions on movement sense during active tasks. For example, a new task modality where participants must detect passive movements while contracting their muscles at controlled intensities might be proposed. This would be interesting since the afferent responses of muscle spindles are more complex and poorly understood during voluntary contractions due to their fusimotor activation^45^.

Although passive movement sense assessment enhances kinesthesia-related fundamental knowledge, the observations made here and in the literature are hardly applicable to daily life or sport. Experiencing slow passive movements in relaxed muscles is indeed far from ecological conditions. Nonetheless, despite the unpractical nature of the task used to evaluate movement sense, our findings open new avenues of reflection on the effects of VA deficits on kinesthesia. For example, persistent VA deficits observed following soccer games^28^ may involve kinesthetic alterations in the ensuing days that may prolong the negative impact on motor performance. Future studies should investigate the effects of ecologically induced VA deficits (e.g., after soccer games and trail running) on movement sense. In addition, to better understand the relationship between movement sense alterations and VA deficits, future studies should also focus on the effects of long-lasting VA deficits on movement sense.

## Conclusion

In summary, we demonstrated that repeated submaximal eccentric contractions led to movement sense alterations only in presence of VA deficits, i.e., only immediately after the eccentric exercise. The present study broadens knowledge about the effects of repeated eccentric contractions on kinesthetic senses, following a previous study on the assessment of position sense using active bilateral joint position matching tasks^19^. Therefore, whatever the assessment modality and the kinesthetic sense evaluated (i.e., active contralateral matching for position sense or passive movement detection for movement sense), deleterious effects of repeated eccentric contractions on kinesthesia have been observed only in the presence of VA deficits. Movement and position sense alterations could therefore result from central factor alterations due to the repetition of eccentric contractions. Future studies are now needed to better understand how eccentric contractions affect the integration and processing of kinesthetic inputs at the somatosensory cortex level.

## Data Availability

The datasets generated during and/or analyzed during the current study are available from the corresponding author on reasonable request.
